# Factors associated with increased risk for pain catastrophizing in patients with chronic neck pain: A retrospective cross-sectional study

**DOI:** 10.1097/MD.0000000000004698

**Published:** 2016-09-16

**Authors:** Sang Jun Park, Rippy Lee, Duck Mi Yoon, Kyung Bong Yoon, Kiwook Kim, Shin Hyung Kim

**Affiliations:** Department of Anesthesiology and Pain Medicine, Anesthesia and Pain Research Institute, Yonsei University College of Medicine, Seoul, Republic of Korea.

**Keywords:** anxiety, catastrophizing, chronic neck pain, depression, insomnia

## Abstract

Pain catastrophizing is becoming increasingly recognized as a clinically important psychological factor in chronic musculoskeletal pain. In this retrospective cross-sectional study, we have identified factors associated with an increased risk for pain catastrophizing in chronic neck pain (CNP) patients. We obtained data from our medical database on 331 patients who were treated for neck pain as their chief complaint at our clinic. The Pain Catastrophizing Scale (PCS) was used to define a high pain catastrophizing state (PCS score ≥21) in this study. Patient demographics, pain-related factors, and psychological factors were evaluated with logistic regression analysis to identify risk factors of high pain catastrophizing among patients with CNP. A total of 256 patients with CNP satisfied the study inclusion criteria and were included in the analyses. The median PCS score was 16 (range, 0–45), and 86 of 256 patients (33.5%) reported a PCS score ≥21. In multivariate analysis, high pain intensity, clinical insomnia, and a high level of depression/anxiety were strongly associated with high pain catastrophizing in patients with CNP. Depression was the strongest predictor of high pain catastrophizing, with an odds ratio of 7.35 (95% confidence interval 2.23–24.22). High pain catastrophizing was not significantly related to age, gender, comorbidities, or neck pain-related physical symptoms. In conclusion, poor psychological states should be addressed as an important part of pain management in CNP patients who are susceptible to high pain catastrophizing.

## 1 Introduction

Chronic neck pain (CNP) is one of the most commonly reported complaints among people with chronic musculoskeletal pain, and has many negative effects on quality of life.^[1,2]^ The prevalence of CNP is increasing, with rising personal, social, and health costs.^[1,2]^ Like other chronic pain conditions, cervical spine studies have shown that CNP is closely related to psychological states such as anxiety, depression, and pain catastrophizing.^[3,4]^

Catastrophizing has been defined as an exaggerated negative mental mindset brought to bear during an actual or anticipated painful experience.^[5]^ It is characterized by the tendency to magnify the threat value of the pain stimulus, feeling helpless in the context of pain, and by a relative inability to inhibit pain-related thoughts in anticipation of, during or following a painful encounter.^[6–7]^ Pain catastrophizing is a key factor that defines how cognition, beliefs, coping strategies, and functioning are related to the experience of pain.^[5–7]^ In fact, catastrophizing thinking seems to play a central role in the development of chronic disabling pain.^[8,9]^ Specifically, previous studies have been reported that pain catastrophizing is associated with increased pain intensity, disability, lower pain threshold/tolerance levels, poor prognosis, and a poor response to pain interventions in neck pain conditions.^[9–13]^

The Pain Catastrophizing Scale (PCS) is a self-report instrument measuring the patient's catastrophizing level, which incorporates items explicitly designed to assess other elements of catastrophizing.^[14]^ Initial factor analytic work indicated that the PCS yielded 3 second-order factors (i.e., helplessness, rumination, and magnification).^[7,14]^ The PCS is considered to be the most comprehensive instrument for this assessment, with good psychometric properties of the catastrophizing construct.^[7,14]^ It has also been validated in a community setting, and a number of studies have replicated this factor structure using confirmatory factor analytic methods in diverse subject groups.^[7,15]^

It is clinically important to identify subgroups of CNP patients with a high pain catastrophizing. This prediction may be important when devising treatment strategies for CNP patients with high pain catastrophizing who are likely to be especially vulnerable to the negative impacts of pain and treatment outcome. However, most pain clinics do not normally have the resources or expertise to provide a detailed pain catastrophizing assessment for CNP patients. The aim of this retrospective, cross-sectional study was to identify factors associated with an increased risk for pain catastrophizing based on PCS, among demographic factors, pain-related factors, and common psychological factors in CNP patients.

## 2 Methods

### 2.1 Study population

This retrospective, cross-sectional study was approved by the institutional review board. The sample population in this study was defined as consecutive CNP patients who received treatment for pain and completed the PCS between January and December 2015 at our outpatient clinic. Data were obtained from a clinical data retrieval system in our institution using neck pain related diagnoses (ICD-10). Neck pain was defined as pain in the anatomical region of the neck with or without radiation to the head, trunk, and upper limbs.^[16]^ The anatomical region of the neck was defined as the posterior neck region from the superior nuchal line to the spine of the scapula, and the side region down to the superior border of the clavicle and the suprasternal notch. Chronicity was established by the persistence of pain beyond 3 months of symptoms. Patients with acute neck pain were excluded (pain duration < 3 months). We excluded patients with current infectious diseases, cancer, and psychiatric and neurologic disorders that would preclude completion of pain-related questionnaires. Patients with major structural pathologies of the cervical spine, including fractures, spinal cord injuries, infections, or neoplasms, were also excluded. Patients were excluded if they reported that their psychological symptoms predated the onset of neck pain by more than 1 month because this study focused on pain-related psychological symptoms, or if they were diagnosed with primary shoulder diseases or peripheral neuropathy.

### 2.2 Pain catastrophizing measurement

The prevalence and severity of pain catastrophizing were assessed using the PCS data recorded at the first-visit interview. The PCS is composed of 13 items measuring catastrophizing thoughts or feelings accompanying the experience of pain.^[14,17]^ Respondents are asked to reflect on past painful experiences and to indicate the degree to which each of the 13 thoughts or feelings were experienced when in pain. Each item is graded on a 5-point scale (0 = not at all to 4 = all the time), so the global score ranges from 0 to 52, with higher scores indicating a greater pain catastrophizing state. For the purpose of this study, high pain catastrophizing was defined as a PCS score ≥21. A PCS cut-off value for identification of high catastrophizing patients varies according to the type of pain conditions or study purpose^[18,19]^; thus, we analyzed the PCS score of all 1422 pain patients who completed the study between January and December 2015 at our clinic. The median PCS score was 16 (range 0–51), and 427 out of 1422 patients (31%) had a score of 21 or greater. We decided to include the 30% to 40% of our study population with the highest PCS scores in the present study. Thus, a PCS score of 21 or greater was chosen for CNP patients to be arbitrarily classified as high pain catastrophizing patients, and this value is similar to a previous study in osteoarthritis patients.^[20]^

### 2.3 Data measures and assessments

Additional patient data were collected, including age, gender, body mass index (BMI), duration of pain, pain score measured on a 0 to 10 numeric rating scale (NRS) (patients were asked to rate the worst pain that they felt during the last 2 weeks), presence of shoulder and/or arm pain (radicular/referred pain), cervical spine surgery history, presence of pain related compensation (workers’ compensation patients and those involved in accidents with insurance coverage/involvement), presence of neck mobility problems (limited active range of motion of the neck due to pain), suspected cervicogenic headache, presence of comorbid musculoskeletal pain conditions (reported pain in 2 or more anatomical areas other than the neck and upper limbs, such as the lower back, lower limbs, and joints), presence of medical comorbidities (diagnosed hypertension, diabetes mellitus, heart diseases or neurologic diseases currently requiring medical treatment), and level of anxiety and depression assessed by the 14-item Hospital Anxiety and Depression Scale (HADS).^[21]^ These 14 items, each scored on a 0 to 3 scale, are used to measure the degree of anxiety (7 items) and depression (7 items). Thus, the 2 subscales range from 0 to 21, with higher scores indicating an increased likelihood of an anxiety or depressive disorder. The cut-off value for the identification of suspected cases is generally considered to be 8 of 21 points.^[21]^ The severity of insomnia was assessed by the 7-item Insomnia Severity Index (ISI).^[22]^ Each item is graded on a 5-point scale (0–4), so the global score ranges from 0 to 28, with higher scores indicating more severe insomnia. According to the recommended score interpretation guidelines, an ISI score of 15 or more is considered clinically significant insomnia.^[22]^ The demographic data and pain-related clinical data, PCS, HADS, and ISI data were obtained from all patients by independent resident doctors during the preliminary medical examination.

### 2.4 Statistical analysis

The minimum sample size for multiple regression analysis was calculated by a power analysis.^[23]^ To be statistically significant in a 95% of power with the anticipated effect size (f^2^) of 0.15, 4 possible predictors, and an alpha level of 0.05 required 129 patients. Continuous variables are shown as mean ± SD, and categorical variables are shown as the number (percentage). Logistic regression was used to compute crude odds ratios (ORs) with 95% confidence intervals (CIs) for variables associated with high pain catastrophizing (PCS score ≥21). The variables used for analysis included demographic data (age, gender, BMI), duration of pain (<1 or ≥1 year), pain score (NRS <7 or ≥7), shoulder/arm pain, spine surgery history, financial compensation, neck mobility problems, headache, medical comorbidities, comorbid musculoskeletal pain, insomnia (ISI <15 or ≥15), and anxiety and depression (HADS <8 or ≥8). For logistic regression analysis, ISI and HADS were used as categorical variables according to the recommended cut-off values with clinical significance. Variables with a *P*-value <0.05 were included in the multivariate logistic regression analysis to estimate adjusted ORs with 95% CIs. Statistical analysis was performed with the Statistical Package for Social Sciences (SPSS, version 20.0; SPSS Inc., Chicago, IL). Values of *P* <0.05 were considered statistically significant.

## 3 Results

**Figure 1:**
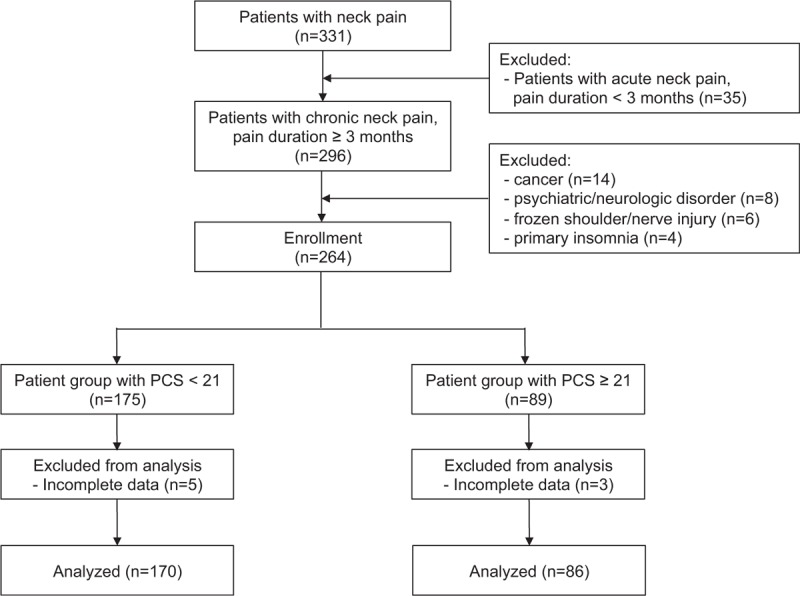
Flow diagram of the study. PCS = Pain Catastrophizing Scale.

Data from 331 patients who were treated for neck pain as their chief complaint at our pain clinic were obtained from electronic medical records. After excluding cases of acute neck pain, a total of 296 patients were evaluated for eligibility. Fourteen patients with diagnosed cancer and eight patients with pre-existing psychiatric and neurologic disorders such as major depressive disorder and Alzheimer disease were excluded. Five patients with suspected primary frozen shoulder and one patient with traumatic brachial plexus injury were excluded. Four patients were excluded for primary insomnia or depressive/anxious symptoms that emerged before neck pain. Eight patients with incomplete data were also excluded. Finally, a total of 256 patients with CNP satisfied the study inclusion criteria and were included in the analyses (Fig. 1).

**Figure 2:**
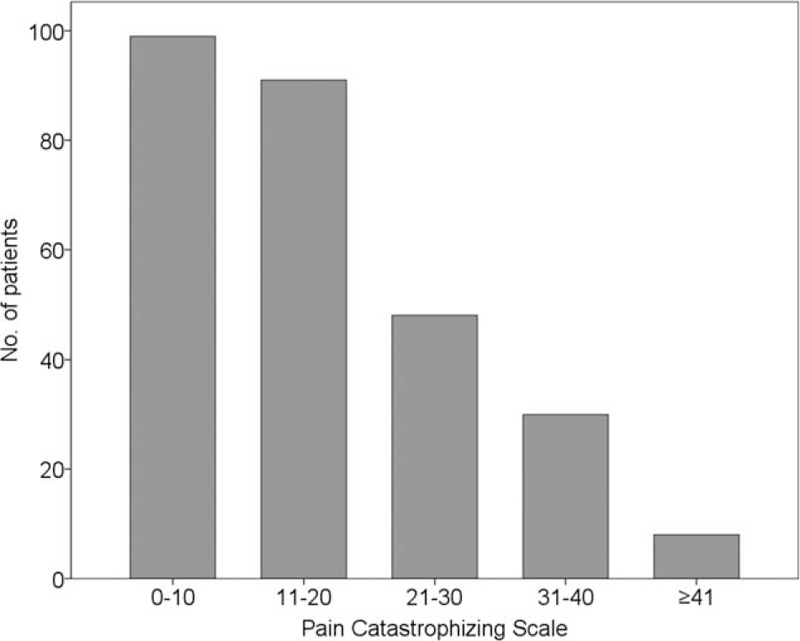
The distribution of Pain Catastrophizing Scale scores in 256 chronic neck pain patients.

Patient demographics and clinical characteristics are summarized in Table 1. Most patients suffered from cervical disc herniation (56.6%) and degenerative spondylosis (34.3%), but a significant number of patients had 2 or more combined cervical spine disease entities. All enrolled patients were taking more than one type of analgesic medication; 66% of patients were taking more than 2 types of analgesic medications. The median PCS score was 16 (range, 0–45), and 86 of 256 patients (33.5%) reported a PCS score ≥21 (Fig. 2).

**Table 1:**
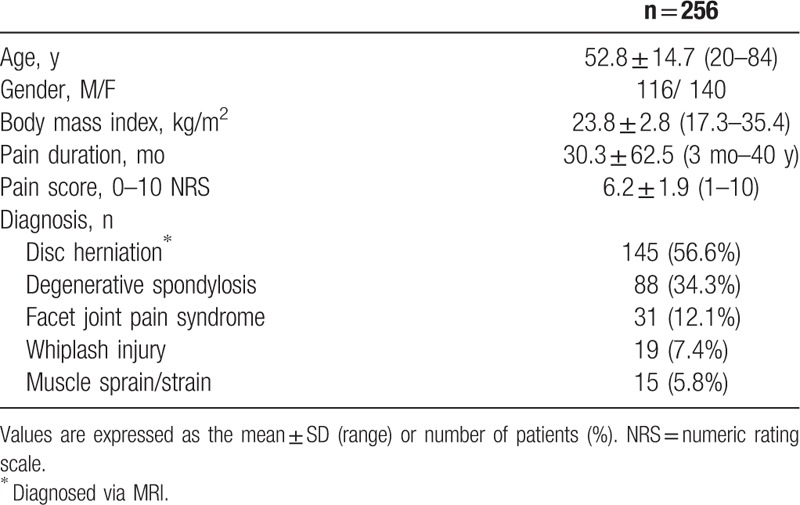
Demographics and clinical characteristics.

In univariate analysis, we found that female gender, high pain score (NRS ≥7), presence of medical comorbidities, presence of comorbid musculoskeletal pain, clinical insomnia (ISI ≥15), and a greater level of anxiety and depression (HADS ≥8) were significantly associated with high pain catastrophizing (Table 2). The incidence of high pain catastrophizing increased gradually with patient age, but the difference was not statistically significant (*P* = 0.24). Patients with a previous spine surgery history had a higher incidence of high pain catastrophizing than those without, but the difference was not statistically significant (*P* = 0.08). Patients involved in pain-related compensation showed a higher incidence of high pain catastrophizing than those without, but there was no statistically significant differences (*P* = 0.09). Common neck pain related symptoms such as shoulder/arm pain, neck mobility problems, and headache showed no significant association with high pain catastrophizing in this study.

**Table 2:**
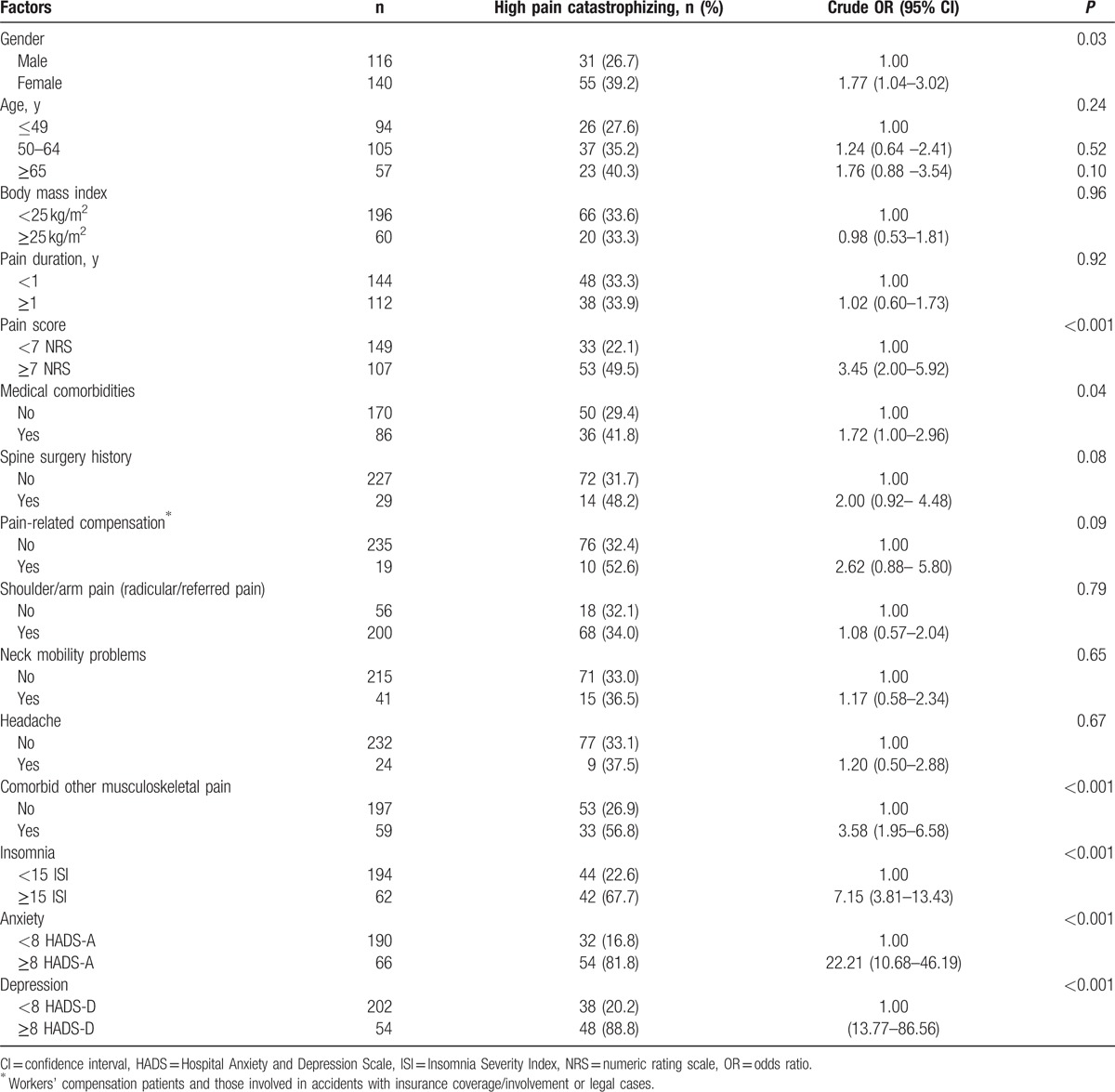
Crude odds ratios for factors associated with high pain catastrophizing (Pain Catastrophizing Scale ≥21) in chronic neck pain: results of univariate analysis.

Multivariate logistic regression analysis revealed that a high pain score (NRS ≥7), clinical insomnia (ISI ≥15), and a greater level of anxiety and depression (HADS ≥8) were significantly associated with high pain catastrophizing in our study population (Table 3). Among the aforementioned variables, a greater level of depression (HADS ≥8) was the strongest determinant predicting high pain catastrophizing, with an OR of 7.35 (95% CI 2.23–24.22). For the test of the goodness of fit of a logistic model, the coefficient of determination (*R*^2^) was 0.447 (*P* < 0.001).

**Table 3:**
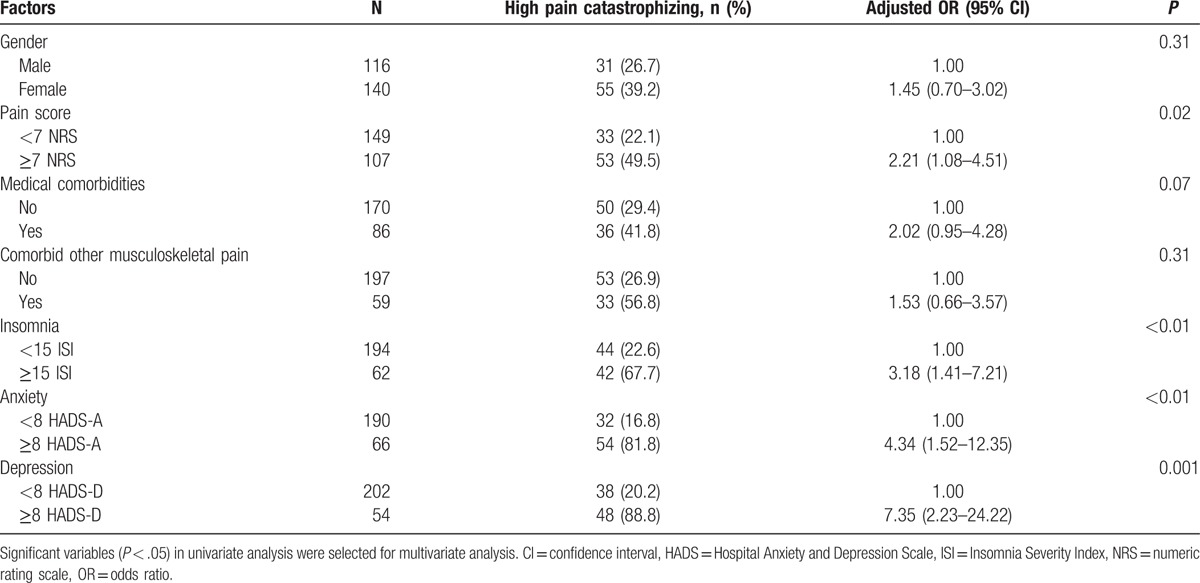
Adjusted odds ratios for factors associated with high pain catastrophizing (Pain Catastrophizing Scale ≥21) in chronic neck pain: results of multivariate logistic analysis.

## 4 Discussion

In the present study, we sought to identify predictors associated with high pain catastrophizing related to demographic factors, pain-related factors, and psychological factors in CNP patients. We found that high pain intensity, clinical insomnia, and a high level of anxiety/depression were strongly associated with high pain catastrophizing in CNP patients.

Centrally mediated psychological symptoms such as anxiety, depression, and sleep disturbance are frequently observed in chronic pain conditions,^[24]^ and these symptoms are closely related to increased pain intensity.^[25]^ Our main finding was that these common psychological factors are independently associated with high pain catastrophizing in CNP patients after adjusting for pain intensity.

Depression was revealed to be the strongest predictor for high pain catastrophizing in CNP patients. Although there is still an ongoing debate on the relationship between catastrophizing and depression in chronic pain,^[26]^ pain catastrophizing accounted for about 40% of the variance in depression severity in a large chronic pain population.^[27]^ Moreover, pain catastrophizing and heightened depressed mood showed an additive and adverse effect on the impact of pain relative to either alone.^[28]^ On the contrary, a recent study demonstrated that the net suppression effects of pain catastrophizing point to anxiety sensitivity, enhancing the effect of catastrophic pain cognition on pain hypervigilance using structural equation modeling in chronic musculoskeletal pain.^[29]^ With regard to insomnia, the probability of pain improvement decreases as baseline sleep quality decreases in neck pain patients.^[30]^ Campbell et al^[31]^ found that pain catastrophizing moderated the relationship between sleep efficiency and central sensitization in chronic pain patients. Collectively, although the interaction between depression, anxiety, insomnia, and pain catastrophizing is complex and not fully understood, these psychological factors seem to significantly influence pain perception in chronic pain patients, directly or indirectly. Furthermore, recent studies increasingly report that improvement in these psychological symptoms as moderators can lead to better treatment outcomes in pain patients.^[32–34]^ Therefore, our results underscore the need to address these psychological symptoms in the management of patients with CNP and may create a pathway for a better-tailored treatment approach for this patient group.

Despite a lack of statistical significance in multivariate analysis, high pain catastrophizing was frequently observed in female patients and in patients with medical comorbidities and comorbid musculoskeletal pain. Female patients are more susceptible than male patients to high pain catastrophizing under chronic pain conditions.^[3]^ However, similar to our results, Hirsh et al^[35]^ reported that there were no gender differences in catastrophizing in adults with physical disability and chronic pain after controlling for other factors. Therefore, our results showed that the presence of poor psychological states may contribute more to pain catastrophizing than gender differences in CNP patients, although high pain catastrophizing is more prevalent in the female gender. On the contrary, patients suffering from medical comorbidities seem to be more prone to poor mental health, emotional distress, and insomnia.^[36]^ Also, Niederstrasser et al^[37]^ demonstrated that pain catastrophizing and fear of pain increased the risk of multisite pain following experimentally induced musculoskeletal injury. Therefore, patients with medical comorbidities or multisite musculoskeletal pain may be closely linked to a high level of pain catastrophizing. In addition, we observed that a high pain catastrophizing is relatively more frequent in older CNP patients who were vulnerable to psychological distress.^[38]^ This age-related tendency also may contribute to high pain catastrophizing in patients with medical comorbidities or multiple pain sites in our population.

Our study has several limitations that suggest directions for future research. First, the study was conducted in a single clinical setting including a selected study population with a homogeneous racial background. Second, our findings are cross-sectional in nature; thus, a causal relationship between pain catastrophizing and current pain or treatments could not be determined definitively. We could not control potential confounders such as the type of current medication and treatment, which could affect pain catastrophizing. Third, the PCS does not contain parameters for measuring the behavioral aspect of pain catastrophizing,^[7]^ which may play an unknown role in determining the severity of catastrophizing. In addition, clinically meaningful PCS scores on pain catastrophizing before and after treatment in CNP have not been clearly determined.^[19]^ Therefore, these limitations of this study should be considered when interpreting the data. Better-controlled and larger studies are needed to evaluate the influence of pain catastrophizing and its correlates on clinical outcomes in CNP patients.

In conclusion, we found that high levels of 3 common psychological symptoms, depression, anxiety, and insomnia, could predict high pain catastrophizing status in the CNP population, even after controlling for pain intensity and various demographic and clinical factors. Therefore, poor psychological states should be addressed as an important part of pain management in CNP patients who are highly susceptible to high pain catastrophizing.
